# Evaluating a School-Based Intervention on Adolescents’ Ethnic-racial Identity in Sweden

**DOI:** 10.1007/s10964-024-02046-y

**Published:** 2024-07-08

**Authors:** Amina K. Abdullahi, Moin Syed, Linda P. Juang, Sofia Berne, C. Philip Hwang, Ann Frisén

**Affiliations:** 1 Department of Psychology, University of Gothenburg, Gothenburg, Sweden; 2 Department of Psychology, University of Minnesota, Minneapolis, MN USA; 3 Department of Inclusive Education, University of Potsdam, Potsdam, Germany

**Keywords:** Ethnic identity, Ethnic-racial identity, Identity process, Identity content, Intervention, Adolescence

## Abstract

Finding developmentally appropriate ways to support youth in understanding their own ethnic-racial identity is needed, particularly in contexts like Sweden where such support is not the norm. This preregistered longitudinal study examined whether an 8-week school-based intervention, the Identity Project, impacted youth ethnic-racial identity exploration (participation and search), resolution, private regard, and centrality. Participants were 509 adolescents in the 10^th^ grade (*M*_*age*_ = 16.28, *SD* = 0.80; 65% self-identified girls; 52% minoritized ethnic background), who were randomized into an intervention or wait-list control group and assessed at baseline and three times post-intervention. The findings indicated an initial and simultaneous effect of the intervention only for exploration participation and resolution but did not show the expected chain of effects with earlier exploration predicting later resolution. Growth models indicated a greater increase in exploration participation over time for the intervention group than the control group. The findings indicate a mixed picture about the effectiveness of the intervention, with effects primarily narrowly targeted to exploration participation, but nevertheless highlight the potential for supporting Swedish youth in engaging with their ethnic-racial identities.

## Introduction

Ethnic-racial identity is a central development task, particularly among minoritized youth in ethno-racially diverse societies (Umaña-Taylor et al., 2018). Much of the knowledge about ethnic-racial identity and how it develops has been conducted in the U.S., and thus there is a need for better understanding of the topic in understudied yet increasingly ethno-racially diverse contexts, such as Sweden. Furthermore, whereas there has been extensive research into the various social contexts and socialization agents that can support ethnic-racial identity development (Ruck et al., 2021), there has been recent interest in facilitating ethnic-racial identity development via active intervention (Umaña-Taylor et al., 2018). Finding developmentally appropriate ways to support youth in understanding their own ethnic-racial identity is needed, particularly in contexts like Sweden where such support is not the norm (Abdullahi et al., 2024). Accordingly, the current study reports on whether an 8-week school-based intervention, the Identity Project (Umaña-Taylor & Douglass, 2016), could promote youth ethnic-racial identity development in Sweden. Moreover, the current study addresses limitations of past evaluations of the Identity Project to inform how to optimally intervene on this important developmental task.

### Ethnic-racial Identity during Adolescence

Although establishing a sense of identity is a critical developmental task across the lifespan (Erikson, 1968), adolescence is a particularly salient time for identity work as youth are increasingly concerned about who they are (Crocetti, 2017). One important domain of youth identity development is ethnic-racial identity, a multidimensional construct that encompasses thoughts, feelings, behaviors, and everyday experiences associated with individuals’ ethnic/racial groups (Umaña-Taylor et al., 2014). Exploration and resolution are two of the central processes through which youth develop their ethnic-racial identities (Umaña-Taylor, Douglass et al., 2018). Exploration pertains to how individuals engage in behaviors that facilitate learning more about their ethnicities and what they might mean for their identities. Exploration comprises two distinct types: participation and search (Syed et al., 2013). *Participation* includes engaging in concrete, active behaviors that lead individuals to feel that they have learned something about their ethnicities. In contrast, *search* consists of behaviors that could be more inward facing (e.g., reading books) and do not necessarily result in youth having learned something specific about the meaning of their ethnicities. The process of resolution refers to the cognitive aspect of ethnic-racial identity, specifically the degree to which youth have a sense of clarity regarding the role of ethnicity for their lives.

Theoretically, positive development of ethnic-racial identity involves attaining a sense of resolution through meaningful exploration, as opposed to gaining resolution solely as a result of ascribed identifications (Umaña-Taylor et al., 2018). This process of exploration allows youth to feel more confident about their sense of resolution, which can help them interact with others in discussions about ethnicity and race (Umaña-Taylor, 2018). The development of ethnic-racial identity also involves dimensions of content, or what the identity actually looks like, such as private regard and centrality (Sellers et al., 1998). Private regard is the extent to which youth have positive feelings about their self-identified group memberships, and centrality is the extent to which ethnicity is an important part of one’s identity.

The collective literature has highlighted the importance of examining both the process and content of ethnic-racial identity, as they are related to clarity in youth’s self-concept as well as to better psychosocial and academic adjustment (Rivas-Drake et al., 2014). These findings about the role and significance of ethnic-racial identity development for the lives of youth bring attention to supporting adolescents’ development toward ethnic-racial identity resolution. This can be done through the family, peer, and schools as part of everyday life (Ruck et al., 2021), but can also be through active intervention designed to enhance the developmental process, such as the Identity Project.

### The Identity Project Intervention

The Identity Project intervention was informed by developmental theory and the accumulated research showcasing general positive links between ethnic-racial identity and psychosocial adjustment (see Umaña-Taylor & Douglass, 2016 for a detailed description). The intervention consists of eight sessions in which youth are encouraged to critically engage in classroom activities and discussions on the social constructs of ethnicity and race. The activities focus on exploration of the potential personal meaning of self-identified group memberships for youth’s ethnic-racial identity. The conceptual model of change proposes that the intervention leads to a chain of effects where it first elicits increased ethnic-racial identity exploration and, through exploration, elicits increased ethnic-racial identity resolution.

The intervention was developed and initially evaluated in the U.S. and included youth with a range of self-identifications (Umaña-Taylor et al., 2018). Participants in the intervention showed an increased level of ethnic-racial identity exploration post-intervention and exploration was in turn associated with a higher level of resolution. The results thus supported the proposed conceptual model of change. Further, minoritized adolescents had a steeper increase in resolution from baseline to the first post-intervention follow-up compared to white students, whereas the latter group had a more positive increase in resolution post-intervention to the one-year follow-up (Sladek et al., 2021). Moreover, the intervention was positively associated with exploration for youth with an average or higher level of baseline centrality but not for youth with below average levels of centrality (Wantchekon et al., 2021).

The effectiveness of the intervention in the U.S. raised questions about whether it may also be effective in other national contexts, particularly in Europe (Juang et al., 2023). European adaptations of the Identity Project have sought to retain its core focus while tailoring it to the particular national context. One reason for doing so is that ethnicity/race-related constructs such as minoritization can have varying connotations across societies. It is, for example, common to report racial identity labels in the U.S. (Sladek et al., 2022), whereas in Germany, Italy, or Sweden minoritization is often overtly expressed in reference to actual or assumed familial migration histories (see Juang et al., 2023). Taken together, the literature suggests that some concepts from the U.S. are applicable to European nations, but that the entire research approach cannot be automatically transferred.

Although most studies on ethnic-racial identity have been conducted in the U.S., the limited studies in European contexts have shown similar patterns of results (Schotte et al., 2018). However, the published evaluations of the intervention in Europe provide mixed evidence for the proposed model of change. The study in Germany indicated that youth ethnic-racial identity exploration increased through the intervention for one of the two participating cohorts, whereas resolution did not increase (Juang et al., 2020). Similarly, in Italy the intervention was associated with increases in exploration, but exploration was not associated with increases in resolution (Ceccon et al., 2023). This mixed support suggests a need to examine the intervention in new contexts, such as Sweden.

The intervention was adapted for a Swedish context by carefully considering the sociohistoric context of Sweden, particularly relating to the concepts of ethnicity and race (Juang et al., 2023). Sociohistoric contextualization is crucial as it has implications for how concepts such as ethnicity, race, and ethnic-racial identity are approached (Umaña-Taylor et al., 2014). There is a general reluctance to discuss the term race in Sweden as it is vernacularly understood through its pseudo-scientific use in the 20^th^ century (von Brömssen, 2021). Further, while race is seldom used as a concept, people are more commonly, and arbitrarily, categorized through the dichotomy of “Swedish” or “immigrant” (Gyberg et al., 2018), where “Swedishness” is often equated with whiteness (Adolfsson, 2024). Moreover, the official definition of ethnicity in Sweden is, “Ethnicity refers to someone belonging to a group of people who have the same national or ethnic origin, skin color, or other similar characteristics. Everyone has at least one ethnicity. People who belong to an ethnic minority often identify as both, for example, Sámi or Chilean and Swedish” (Equality ombudsman, 2024). Thus, racialized phenotypic characteristics typically assigned to the concept race are to some extent incorporated in ethnicity in the Swedish context.

Indeed, a major change in the Swedish adaptation of the intervention was the use of the main concept ethnic identity rather than ethnic-racial identity. Nevertheless, discussions on race and racism in relation to ethnicity were included. Further adaptations included changing the sociohistoric setting of intervention activities to Sweden; for example, discussing stories of discrimination from Swedish archives, or discussing video recorded interviews with young adults with varying self-identifications in Sweden who reflected on the meaning of their ethnic-racial identities. All activities and lesson goals of the original intervention were retained although their specific content differed from the U.S. version (see Juang et al., 2023).

### Improving Upon Limitations of Previous Evaluation Studies

Extending the intervention to the Swedish context is valuable on its own, but the current study also sought to improve upon the existing literature. This is needed given the promising, albeit mixed results of prior adaptations. The current study addressed five limitations in the previous studies. First, early versions of the conceptual model for the Identity Project proposed that the intervention leads to post-intervention (T1) increases in both exploration participation and resolution, which subsequently should be positively associated with later resolution (T2; Umaña-Taylor & Douglass, 2016). However, empirical studies concerning intervention effects have tested a conceptual model where the intervention leads to T1 exploration participation, which then is positively associated with T2 resolution (Umaña-Taylor et al., 2018). These studies have not controlled for T1 resolution, which is important because the intervention could already have had a direct effect on resolution at this time point. If unaccounted for, T1 resolution could be a confounding variable in relation to later resolution (T2), and so the relationship between T1 exploration participation and T2 resolution may be exaggerated. Thus, accounting for prior resolution would enable a more stringent assessment of whether exploration is the key to later resolution. Second, prior studies have conceptualized and measured intervention effects on exploration by focusing on participation and not search (e.g., Ceccon et al., 2023). Including both forms of exploration is motivated by their embeddedness in the developmental theory of ethnic identity (Syed et al, 2013), and addresses whether the intervention only targets participation, which it was designed to increase, or includes search as well, and thus impacts ethnic-racial identity exploration broadly. Third, there is a limited understanding of how key study variables change over time (Sladek et al., 2021). Assessing trajectories of change goes beyond the modeled interrelatedness of variables in the intervention’s conceptual model and enables a separate examination of how the intervention acts on each of the key variables over time. Fourth, the intervention has mainly been evaluated based on the explicit intervention aims of affecting process components (for an exception see Wantchekon et al., 2021). Although the intervention makes no explicit claims to affect content components, it arguably contains activities that may impact ethnic-racial identity content, such as critical discussions on ethnicity- or race-related stereotypes, discrimination, historic and/or contemporary societal injustices, with the goal of providing youth with a space to discuss and question such expressions. Including both process and content components (e.g., centrality and private regard) in the study of ethnic-racial identity allows for more holistic, and specific, assessment of potential intervention effects on ethnic-racial identity dimensions. Nevertheless, since the intervention is not designed to target private regard or centrality, both are included in this study on an exploratory basis. Finally, reported limitations of prior studies include small sample sizes (Umaña-Taylor et al., 2018) and limited statistical power for detecting intervention effects (Juang et al., 2020).

## The Current Study

Given the importance of finding effective ways to support youth ethnic-racial identity development in ethno-racially diverse societies, the current preregistered study examined whether the Identity Project intervention positively impacted ethnic-racial identity process and content components among 10^th^ grade students in Sweden. The first research question concerned whether the intervention a) prompted the process of ethnic-racial identity exploration (participation and search) and b) through exploration positively affected the process of ethnic-racial identity resolution. The intervention was hypothesized to be positively associated with post-intervention (T1) exploration (participation and search), which in turn would be positively associated with post-intervention (T2) resolution, while accounting for post-intervention (T1) resolution. The role of content components private regard and centrality in the intervention’s conceptual model of change was explored in relation to this question. The second question was exploratory and concerned how the process components participation, search, and resolution changed over time, and potentially as an effect of the intervention. All process components were expected to be positively affected by the intervention. How the content components private regard and centrality changed over time and as an effect of the intervention was explored in relation to this question. The third question was exploratory and concerned whether the intervention had differential effects on ethnic-racial identity components based on whether students were minoritized or majoritized.

## Method

### Participants

Participants were 509 adolescents (*M*_*age*_ = 16.28, *SD* = 0.80) in 10^th^ grade across four high schools. 52% of adolescents (*n* = *266*) were themselves or (all) their parents born outside of Sweden i.e., minoritized, and 40% (*n* = *205*) were born in Sweden and had at least one parent born in Sweden i.e. majoritized, and were thus grouped in accordance with Statistics Sweden (2022). An additional 8% (*n* = 43) were missing on the minoritization item due to non-response or absence. 61% of adolescents attended theoretical education that prepares students for university and 39% attended vocational education that qualifies youth for work within their educational field after high school. 65% identified as girls, 26% as boys, 1% did not identify with either of these categories, and 8% were missing on the gender item due to non-response or absence.

### Procedure

The Swedish Ethical Review Authority approved the design and data collection of this study (Dnr 2021-04027). High schools that according to the Swedish National Agency for Education’s (2020) official records reported that an average of 40% or more of their students were minoritized, in two cities in the southwest, were eligible for inclusion. Principals in 19 high schools were contacted through an e-mail with information about the study, which was followed up with a phone call. Eight schools declined participation and seven did not respond. Principals in four high schools agreed to let their schools participate and accepted the offer of an in-school information meeting in which a research assistant and/or PhD student informed staff about the study. Staff received information about the study digitally prior to this meeting. Teachers were asked to let the researchers approach their students with a request for participation in the study. Invitations and an information letter about the study were forwarded to students by their teachers. The information letter clarified that participation was voluntary, could be discontinued at any time, and that answers would be treated anonymously. Further, it informed students about how they could confirm their consent to be part of the study (beginning of the online questionnaire). Moreover, it clarified that the intervention lessons were a part of the regular school curriculum and that the data collection through surveys was a part of the research study. Thus, students could partake in the intervention and choose to not participate in the study. In accordance with the Swedish Ethical Review Act (SFS 2003:460), parental consent was not required or requested due to all students being over 15 years of age.

10^th^ grade classes (*N* = 22), from the four schools, were randomly allocated to an intervention or a wait-list control group respectively, resulting in 290 students in the intervention group and 219 students in the control group. If a school for example had four classes in the social science program and four in the natural science program, two of each type of program would be randomized for intervention in the fall semester and two in the spring semester respectively. The intervention and control groups did not differ regarding minoritization (χ^2^(1) = *1.95*, *p* = 0.21), gender (χ^2^(2) = *0.63*, *p* = 0.73*)*, type of education (χ^2^(1) = 2.26, *p* = *0*.13), or SES (χ^2^(360) = 0.68, *p* = 0.50). The groups differed in age (*t*(450) = −2.92, *p* = 0.005, *d* = −0.27, Intervention: *M* = 16.37, *SD* = 0.85, Control: *M* = 16.16, *SD* = 0.73).

The eight intervention lessons (1 h/lesson) were administered to the intervention group over a period of 10 weeks in the fall semester of 2021 and to the wait-list control group over a period of nine weeks in the spring semester of 2022. Time periods varied due to holidays and rescheduled classes due to absences during the Covid-19 pandemic. High schools in Sweden had reopened during this academic year. The control group attended their regular classes prior to partaking in the intervention. Four moderators who were all clinical psychologists (one research assistant, two PhD students, and one PhD) implemented the intervention, with two moderators in each classroom. Of the four moderators, one had a minoritized ethnic background, two had a majoritized ethnic background, and one had both a minoritized and majoritized ethnic background. In all but one class moderators were teamed up so that a majoritized and minoritized ethnic background was represented.

### Data Collection

Data on key study variables were collected through a digitally administered survey during regular school hours, on four occasions over the academic year. Pre-intervention measures (T0) were collected for all students one week prior to the beginning of intervention in the fall semester. Post intervention measures were collected 12 weeks (T1), 16 weeks (T2), and 25 weeks (T3) after baseline measure. The time points were chosen to be as similar as possible to the original U.S. intervention study (Umaña-Taylor et al., 2018). The intervention group participated in the intervention between T0 and T1 and the control group between T2 and T3. Thus, the intervention and control group were comparable at T0, T1 and T2. At T3 the control group had also received the intervention. During data collection, the moderators informed students about the study both in writing and verbally, including information on consent, and could explain important terminology and answer questions about the survey.

### Measures

Demographic variables age, gender, place of birth, parental place of birth and parental educational level were collected at baseline. Minoritization was dummy coded so that 0 = minoritized ethnic background and 1 = majoritized ethnic background. Gender was dummy coded 0 = boy, 1 = girl and 2 = “that division does not suit me”. Type of education was dummy coded so that 0 = theoretical education and 1 = vocational education. SES was calculated using the average score of parental educational level, 1 = No education, 2 = lower secondary school, 3 = upper secondary school, and 4 = College/University, using the relevant Swedish terminology. If students reported a response for one parent, this response was used (Hollingshead, 1971). For each of the key study measures below, reliability estimates were calculated using Cronbach’s α and the mean inter-item correlation (IIC; see Table 1). While Cronbach’s α tends to disfavor shorter scales, the IIC is not as sensitive to the number of items (Streiner, 2003). Higher values on each of the estimates reflects higher internal consistency. However, an IIC between 0.15 and 0.50 is acceptable.

**Table 1:** Bivariate correlations for key study variables, reliabilities, means, standard deviations, and number of participants for intervention (above diagonal) and control group (below diagonal)

		1	2	3	4	5	6	7	8	9	10	11	12	13	14	15	16	17	18	19	20
1	T0 Exploration P	–	0.48***	0.42***	0.41***	0.44***	0.36***	0.35***	0.30***	0.50***	0.30***	0.32***	0.29***	0.26***	0.20**	0.30***	0.22**	0.40***	0.39***	0.30***	0.24***
2	T1 Exploration P	0.49**	–	0.54***	0.47***	0.38***	0.41***	0.36***	0.41***	0.33***	0.51***	0.38***	0.26***	0.31***	0.35***	0.27***	0.23**	0.25***	0.40***	0.23**	0.21**
3	T2 Exploration P	0.39***	0.56***	–	0.52***	0.45***	0.48***	0.49***	0.40***	0.31***	0.32***	0.59***	0.31***	0.30***	0.26***	0.43***	0.30***	0.22**	0.31***	0.36***	0.21**
4	T3 Exploration P	0.35***	0.52***	0.51***	–	0.38***	0.40***	0.45***	0.39***	0.24***	0.25***	0.32***	0.52***	0.31***	0.27***	0.29***	0.42***	0.29***	0.28***	0.22**	0.25***
5	T0 Resolution	0.42***	0.39***	0.32***	0.43***	–	0.43***	0.42***	0.47***	0.33***	0.25***	0.38***	0.26***	0.33***	0.26***	0.31***	0.29***	0.43***	0.38***	0.34***	0.33***
6	T1 Resolution	0.34***	0.47***	0.45***	0.39***	0.52***	–	0.45***	0.45***	0.27***	0.34***	0.34***	0.19**	0.29***	0.38***	0.28***	0.24**	0.35***	0.48***	0.40***	0.35***
7	T2 Resolution	0.51***	0.51***	0.53***	0.45***	0.59***	0.55***	–	0.51***	0.25***	0.29***	0.31***	0.23**	0.19**	0.31***	0.27***	0.32***	0.33***	0.33***	0.34***	0.30***
8	T3 Resolution	0.37***	0.27**	0.26**	0.39***	0.47***	0.54***	0.61***	–	0.35***	0.37***	0.36***	0.24***	0.19**	0.39***	0.27***	0.27***	0.37***	0.39***	0.33***	0.32***
9	T0 Exploration S	0.47***	0.29***	0.20**	0.21**	0.26***	0.22**	0.40***	0.33***	–	0.43***	0.43***	0.35***	0.37***	0.25***	0.29***	0.20**	0.40***	0.35***	0.23**	0.22**
10	T1 Exploration S	0.32***	0.49***	0.27***	0.31***	0.27***	0.24**	0.41***	0.20*	0.55***	–	0.53***	0.41***	0.44***	0.48***	0.33***	0.29***	0.20**	0.37***	0.15*	0.16*
11	T2 Exploration S	0.34***	0.34***	0.49***	0.37***	0.29***	0.26**	0.38***	0.22**	0.52***	0.57***	–	0.50***	0.52***	0.39***	0.61***	0.41***	0.30***	0.36***	0.38***	0.24**
12	T3 Exploration S	0.28***	0.29***	0.39***	0.55***	0.29***	0.25**	0.31***	0.23**	0.47***	0.58***	0.59***	–	0.39***	0.40***	0.52***	0.58***	0.25***	0.26***	0.26***	0.37***
13	T0 Private R.	0.29***	0.40***	0.35***	0.38***	0.20**	0.31***	0.34***	0.26**	0.36***	0.39***	0.48***	0.38***	–	0.50***	0.52***	0.45***	0.35***	0.31***	0.26***	0.27***
14	T1 Private R.	0.26**	0.45***	0.34***	0.26**	0.21**	0.31***	0.33***	0.22**	0.31***	0.57***	0.37***	0.47***	0.61***	–	0.57***	0.60***	0.28***	0.41***	0.32***	0.34***
15	T2 Private R.	0.26**	0.40***	0.36***	0.42***	0.30***	0.31***	0.33***	0.22**	0.34***	0.47***	0.60***	0.58**	0.61**	0.63**	–	0.68***	0.24***	0.32***	0.49***	0.37***
16	T3 Private R.	0.20*	0.16*	0.31***	0.31***	0.24**	0.19*	0.13	0.19*	0.22**	0.18*	0.25**	0.47***	0.46***	0.41***	0.55***	–	0.28***	0.32***	0.38***	0.50***
17	T0 Centrality	0.37***	0.26**	0.23**	0.24**	0.45***	0.43***	0.48***	0.39***	0.32***	0.22**	0.25**	0.13	0.35***	0.24**	0.24**	0.05	–	0.54***	0.54***	0.51***
18	T1 Centrality	0.33***	0.26***	0.23**	0.30***	0.42***	0.46***	0.41***	0.40***	0.26***	0.28***	0.22**	0.29***	0.40***	0.39***	0.25**	0.18*	0.73***	–	0.60***	0.55***
19	T2 Centrality	0.21**	0.28***	0.28***	0.34***	0.44***	0.35***	0.45***	0.36***	0.23**	0.23**	0.30***	0.24**	0.37***	0.30***	0.36***	0.22**	0.65***	0.64***	–	0.56***
20	T3 Centrality	0.20*	0.03	0.20*	0.17*	0.37***	0.30***	0.30***	0.37***	0.15	0.05	0.23**	0.18*	0.22**	0.12	0.23**	0.36***	0.50***	0.49***	0.63***	–
	α	0.73	0.76	0.81	0.80	0.84	0.85	0.87	0.88	0.70	0.76	0.79	0.80	0.76	0.79	0.84	0.84	0.65	0.65	0.52	0.53
	IIC	0.48	0.51	0.59	0.58	0.63	0.65	0.69	0.70	0.32	0.38	0.43	0.44	0.37	0.40	0.48	0.48	0.20	0.19	0.13	0.13
	Interv. *M* (SD)	2.30 (0.88)	2.70 (0.78)	2.76 (0.84)	2.76 (0.80)	3.20 (0.70)	3.31 (0.58)	3.31 (0.66)	3.29 (0.66)	2.55 (0.66)	2.64 (0.66)	2.69 (0.73)	2.76 (0.64)	5.79 (0.85)	5.75 (0.92)	5.67 (1.00)	5.62 (1.05)	4.20 (0.94)	4.31 (0.90)	4.28 (0.84)	4.30 (0.74)
	*n*	272	240	234	232	272	242	236	234	271	241	232	229	270	240	232	232	271	241	234	232
	Contr. *M* (SD)	2.54 (0.83)	2.63 (0.78)	2.72 (0.77)	2.96 (0.74)	3.19 (0.65)	3.15 (0.70)	3.23 (0.66)	3.33 (0.64)	2.65 (0.66)	2.64 (0.69)	2.82 (0.61)	2.81 (0.72)	5.73 (0.92)	5.66 (0.99)	5.60 (1.10)	5.56 (1.15)	4.23 (0.92)	4.11 (0.91)	4.25 (0.72)	4.36 (0.84)
	*n*	195	191	195	173	196	191	196	173	194	191	195	169	194	190	194	167	194	190	194	167

*T* time, *Exploration P* exploration participation, *Exploration S* exploration search, *Private R* private regard, *IIC* inter-item correlation, *Interv* intervention group, *Contr* control group

*p < 0.05; **p < 0.01; ***p < 0.001

#### Exploration Participation

Participation was measured using the three-item exploration subscale of the Ethnic Identity Scale- Brief (EIS-B, Douglass & Umaña-Taylor, 2015). Items were scored on a 4-point Likert scale ranging from 1 (does not describe me at all) to 4 (describes me very well) and were averaged to indicate that higher scores correspond to higher levels of exploration participation. An example item was, “I have participated in activities that have taught me about my ethnicity”. The Cronbach’s alphas for the scale varied between 0.73 and 0.81 across time points. Confirmatory factor analyses for exploration participation (Supplementary Tables S1–S3, all supplementary tables, figures, and analyses are found using the following link: https://osf.io/fhydw) showed acceptable fit indices. The scale showed metric and scalar invariance over time and across minoritized and majoritized youth. It had partial strict scalar invariance as residuals could be constrained to be equal across the two groups for each time point.

#### Exploration Search

Search was measured using the five-item exploration subscale of the Multigroup Ethnic Identity Measure (MEIM, Phinney, 1992). Items were scored on a 4-point Likert scale, ranging from 4 (*Strongly agree*) to 1 (*Strongly disagree*). The items were reverse coded and then averaged to indicate that higher scores correspond to higher levels of exploration search. An example item was, “I think a lot about how my life will be affected by my ethnic group membership”. The Cronbach’s alphas for the scale varied between 0.70 and 0.80 across time points. Confirmatory factor analyses for exploration search (supplementary Tables S4–S6) showed sub-optimal fit indices. The scale showed metric but not scalar invariance over time and across minoritized and majoritized youth groups.

#### Resolution

Resolution was measured using the three-item resolution subscale of the EIS-B (Douglass & Umaña-Taylor, 2015). Items were scored on a 4-point Likert scale ranging from 1 (*does not describe me at all)* to 4 (*describes me very well)* and were averaged to indicate that higher scores correspond to higher levels of resolution. An example item was, “I have a clear sense of what my ethnicity means to me”. The Cronbach’s alphas for the scale varied between 0.84 and 0.88 across time points. Confirmatory factor analyses for resolution (Supplementary Table S7–S9) showed good fit indices. The scale showed metric, scalar and strict scalar invariance over time and across minoritized and majoritized groups.

#### Private Regard

Private Regard was measured using the six-item private regard subscale of the Multidimensional Inventory of Black Identity (MIBI, Sellers et al., 1997). “My ethnicity/my ethnic group(s)” was used in items instead of “Black” to be relevant for students regardless of their ethnic-racial identities (Wantchekon et al., 2021). Items were scored on a 7-point Likert scale ranging from 1 (*strongly disagree*) to 7 (*strongly agree*). One item was reverse coded, and all items were then averaged to indicate that higher scores correspond to higher levels of private regard. An example item was, “I’m happy that I am a member of my ethnic group(s)”. The Cronbach’s alphas for the scale varied between 0.76 and 0.84 across time points. Confirmatory factor analyses for private regard (Supplementary Tables S10–S12) showed poor fit indices. The scale showed metric but not scalar invariance over time and across groups.

#### Centrality

Centrality was measured using the eight-item centrality subscale of the MIBI (Sellers et al., 1997). Items were scored on a 7-point Likert scale ranging from 1 (*strongly disagree*) to 7 (*strongly agree*). Three items were reverse coded, and items were then averaged to indicate that higher scores correspond to higher levels of centrality. An example item was, “My ethnicity is an important reflection of who I am”. The Cronbach’s alphas for the scale varied between 0.52 and 0.65 across time points. Confirmatory factor analyses for centrality (Supplementary Tables S13–S15) showed poor fit indices. The scale showed metric but not scalar invariance over time and across groups.

### Analytic Approach

The study was preregistered (https://osf.io/h2pc6) post data collection but prior to data assessment or analyses. All reported analyses were preregistered unless otherwise specified. Data were checked for skewness and kurtosis and were not altered. Descriptive statistics and bivariate correlations were estimated (Table 1). Baseline comparisons of demographic and key study variables were made between the intervention and control group. Since the intervention was implemented at the classroom level, the 22 classrooms could be an important random factor to control for. However, intra-class correlations for the study variables were all below 0.05 and design effects were below 2 (Supplementary Table S16; Muthén and Satorra, 1995). Thus, classroom nesting was not included in this study. Furthermore, the intervention was not implemented at the school level, and only four schools participated, limiting the potential role of schools as a meaningful random factor (Austin & Leckie, 2018). Thus, school level clustering was not included in analyses.

Path analysis was used to examine the first research question concerning if the Identity Project intervention prompted a sequential increase in the ethnic-racial identity processes exploration and resolution. Next, a series of pre-registered alternative models that, for example, reversed the postulated direction of intervention effects, controlled for post-intervention (T1) resolution, included exploration search, and finally, explored the potential role of content components in the conceptual model of change, were examined (see preregistration and supplementary materials for details on all path models). The path models included concurrent correlations among variables and there were no constraints placed on paths over time.

The pre-registration plan for path models included a contingency for how to deal with potential baseline differences in key process components exploration and resolution by accounting for them as controls. However, due to a baseline difference in age between the intervention and control group, and due to type of education (theoretical or vocational) being associated with post-intervention T2 missingness, robustness checks were conducted for all path models by controlling for age and type of education. Controlling for these variables did not alter the pattern of effects, with one exception. When controlling for age and type of education a previously non-significant association between the intervention and private regard became significant. However, due to reliability issues with the private regard scale, it is difficult to draw inferences from this finding. Thus, all robustness checks for path models are reported in the supplementary materials (https://osf.io/fhydw). Note that the number of participants in the path models are smaller when including controls that have missingness compared to those that only include intervention as an exogenous variable.

Growth curve analysis was used to examine the second research question regarding how the study variables changed over time, and if they changed as an effect of the intervention. Maximum likelihood with robust standard errors was used and analyses adhered to the intention-to-treat principle. The adequate structural trajectory for each variable was established through nested model comparisons of unconditional linear and quadratic latent growth models. These models were compared using model fit indices and chi-square difference tests. The residuals in the growth models were only constrained to be equal over time if the utilized measures had strict scalar invariance over time. Conditional latent growth curve models tested whether the intervention had an effect on the intercepts and slopes of each of the study variables. The factor loadings for the intercept were set to 1, and the factor loadings for the linear slope were set to 0, 3, 4, and 6.25 to reflect the number of months between pre-intervention measure T0 and post-intervention measures T1, T2 and T3 respectively.

Importantly, the wait-list control group received the intervention between post-intervention T2 and T3 and had therefore also become an intervention group at the last wave of data collection. Thus, the wait-list control group’s last measurement (T3) was treated as missing and was estimated using full information maximum likelihood (Wothke, 2000). This allowed for a comparison between an intervention group and a control group that had not received the intervention and was motivated by the focus of research questions on the potential effect of the intervention (as compared to a control) on key variables over time. However, due to this non-random missingness, the initial unconditional growth curve models that did not include intervention could potentially include bias. The intervention would have to be accounted for in the models to get a more reliable examination of best functional fit. Thus, conditional linear and conditional quadratic models that included intervention as a predictor of intercepts and slopes were also compared to confirm that the adequate structural trajectory had been established. Robustness checks were conducted using only the first three waves of data, when the intervention and control groups were comparable. Furthermore, robustness checks controlling for age and type of education were also conducted for all growth curve analyses using three and four waves of data respectively. Results from these robustness checks aligned with reported results in the current manuscript, and they are thus included in the supplementary materials (https://osf.io/fhydw).

To examine the third research question regarding differential effects of the intervention based on minoritization, multigroup path models grouped by minoritization were run for the path models. Unconstrained and constrained multigroup path models were compared using a chi-square difference test. In the case of inequivalent path models, ad-hoc Wald χ^2^ test was used to identify which specific paths varied for minoritized and majoritized youth. To assess potential differential effects of the intervention on trajectories of change based on minoritization, conditional multigroup growth models grouped by minoritization were run. Here, unconstrained models and models that constrained the intervention effect on intercepts and slopes to be equal for minoritized and majoritized youth were compared using a chi-square difference test.

The goodness of fit for path models and growth models respectively were assessed using Chi-square (χ^2^) test of model fit, comparative fit index (CFI), Tucker-Lewis index (TLI), the root mean square error of approximation (RMSEA), and the standardized root mean square residual (SRMR). Models were considered to have good fit to data if χ^2^ test was non-significant, CFI and TLI were equal to or greater than 0.95, RMSEA was below 0.06 and SRMR was below 0.08 (Hu & Bentler, 1999). The path analyses and latent growth curve analyses were conducted using the lavaan package version 0.6–16 in R, (Rosseel, 2012). Sample size estimates can differ between models as they include different exogenous variables that include missing values. Importantly, only nested models were compared using likelihood ratio test.

## Results

Descriptive statistics and preliminary baseline analyses are first presented. Then, path models that examine the conceptual model are presented, with a focus on process then content components. Next, the trajectories of change for each of the key study variables are presented. The potential differential effect of the intervention based on minoritization is reported for the models throughout.

### Descriptive Statistics and Preliminary Analyses

Table 1 shows descriptive statistics, reliabilities, bivariate correlations for key study variables, and the number of students who filled out the survey at each time point. The analytic sample consisted of 509 students. Missing data for the whole sample included students who were absent at data collection or who did not fill out the survey. For the study variables, there were between 8% and 22% missing data (all items missing on a variable) from T0 (baseline) to T3 respectively. Missing data for individual items on study variables was low. Less than 2% of participants had partial responses with 50% or more missing items per variable. Thus, scores were aggregated as means. In consequent analyses, missing data were handled using full information maximum likelihood.

Chi-square tests were conducted to assess whether missingness was related to any of the demographic variables. Missingness was not related to minoritization, gender, or socioeconomic status. However, missingness was related to age at all time-points and was more likely for students in vocational education at T2 and T3 (see https://osf.io/fhydw for more details on missingness). Thus, age and type of education were included as controls in the robustness checks of all analyses.

Concerning the key study variables, the randomized intervention and control groups did not differ on levels of exploration search, resolution, private regard, or centrality. The control group reported a higher baseline level of exploration participation than the intervention group (*t*(465) = 2.87, *p* = 0.004, Cohen’s *d* = 0.27). Further, minoritized youth scored higher than majoritized youth on baseline exploration participation (*t*(465) = 6.77, *p* = <0.001, Cohen’s *d* = 0.63), exploration search (*t*(463) = 6.32, *p* = <0.001, Cohen’s *d* = 0.59), resolution (*t*(466) = 5.89, *p* = <0.001, Cohen’s *d* = 0.55), private regard (*t*(462) = 2.33, *p* = 0.01, Cohen’s *d* = 0.22), and centrality (*t*(463) = 6.17, *p* = <0.001, Cohen’s *d* = 0.58).

### Process and Content Components in the Conceptual Model of Change

A replication model proposing that the intervention prompts T1 exploration participation, which in turn is associated with higher T2 resolution, was a good fit to data (Supplementary Fig. S1; Table 2). The intervention was not associated with T1 exploration participation (*b* = 0.04, *SE* = 0.08, *p* = 0.35). However, T1 exploration participation was positively associated with T2 resolution (*b* = 0.36, *SE* = 0.04, *p* < 0.001). The replication model was then run as a multigroup model, grouped by minoritization. A nested model comparison of an unconstrained model and a model in which all paths were constrained to be equal for minoritized and majoritized adolescents showed no difference (Table 3).

**Table 2:** Fit indices for path models

Model	*N*	χ^2^	df	CFI	TLI	RMSEA (90% CI)	SRMR	AIC	BIC
S1	509	0.78	1	1.00	1.01	0.00 (0.00–0.11)	0.01	1835.18	1860.58
S2	467	4.95*	1	0.98	0.89	0.09 (0.03–0.18)	0.03	1553.61	1586.78
S3	509	0.23	1	1.00	1.06	0.00 (0.00–0.09)	0.01	1846.18	1871.58
S4	467	0.56	1	1.00	1.02	0.00 (0.00–0.11)	0.01	1594.74	1627.91
S5/Fig. 1	467	0.27	1	1.00	1.02	0.00 (0.00–0.10)	0.00	2117.65	2175.69
S6	467	0.23	1	1.00	1.02	0.00 (0.00–0.10)	0.00	2827.99	2915.06
S7	467	8.82	4	0.99	0.97	0.05 (0.00–0.10)	0.03	3925.69	4033.49
S8	467	20.28*	9	0.99	0.96	0.05 (0.02–0.08)	0.03	5593.95	5759.80

See Supplementary Table S19 in supplementary materials for an overview of all fit indices for all analyzed models in the study.

*CFI* comparative fit index, *TLI* Tucker-Lewis index, *RMSEA* root mean square error of approximation, *SRMR* standardized root mean square residual, *CI* Confidence interval*, AIC* Akaike information criterion, *BIC* Bayesian information criterion

*p < 0.05

**Table 3:** Fit indices and model comparison of multigroup path models grouped by minoritization

Model	*N*	χ^2^	df	χ^2^diff	Δ*df*	*p*	CFI	TLI	RMSEA (90% CI)	SRMR	AIC	BIC
S1 Unconstrained	471	1.59	2				1.00	1.03	0.00 (0.00–0.12)	0.02	1634.19	1684.05
S1 Constrained	471	2.40	4	0.80	2	0.67	1.00	1.06	0.00 (0.00–0.08)	0.03	1630.99	1672.54
S2 Unconstrained	467	3.33	2				0.99	0.95	0.05 (0.00–0.15)	0.02	1538.59	1604.93
S2 Constrained	467	5.45	6	2.11	4	0.71	1.00	1.01	0.00 (0.00–0.08)	0.04	1532.70	1582.46
S3 Unconstrained	471	2.26	2				0.99	0.97	0.02 (0.00–0.13)	0.02	1660.28	1710.14
S3 Constrained	471	2.72	4	0.47	2	0.79	1.00	1.07	0.00 (0.00–0.08)	0.02	1656.74	1698.29
S4 Unconstrained	467	2.51	2				1.00	0.97	0.03 (0.00–0.14)	0.02	1589.95	1656.29
S4 Constrained	467	4.39	6	1.89	4	0.76	1.00	1.03	0.00 (0.00–0.08)	0.03	1583.83	1633.59
S5 / Fig. 1 Unconstrained	467	0.29	2				1.00	1.05	0.00 (0.00–0.07)	0.01	2112.43	2228.52
S5 / Fig. 1 Constrained	467	3.73	9	3.44	7	0.84	1.00	1.03	0.00 (0.00–0.02)	0.02	2101.86	2188.94
S6 Unconstrained	467	0.28	2				1.00	1.06	0.00 (0.00–0.07)	0.00	2816.47	2990.61
S6 Constrained	467	6.59	12	6.31	10	0.79	1.00	1.03	0.00 (0.00–0.03)	0.02	2802.78	2935.46
S7 Unconstrained	467	11.88	8				1.00	0.97	0.05 (0.00–0.10)	0.03	3911.75	4127.36
S7 Constrained	467	33.79*	20	21.91	12	0.04*	0.98	0.96	0.05 (0.02–0.09)	0.02	3909.66	4075.51
S8 Unconstrained	467	28.83	18				0.99	0.96	0.05 (0.00–0.08)	0.03	5566.32	5898.02
S8 Constrained	467	57.22*	35	28.39	17	0.04*	0.98	0.96	0.05 (0.03–0.08)	0.05	5560.71	5821.93

Group coded as 0 = minoritized and 1 = majoritized.

See Supplementary Table S20 in supplementary materials for fit indices for all examined multigroup path models

*Constrained*, equality constraints on all paths for minoritized and majoritized youth. *χ2diff*, nested χ2 difference; *CFI* comparative fit index, *TLI* Tucker-Lewis index, *RMSEA* root mean square error of approximation, *SRMR* standardized root mean square residual, *CI* Confidence interval*, AIC*, Akaike information criterion; *BIC*, Bayesian information criterion

*p < 0.05

The replication model was run again, this time controlling for the intervention and control groups’ baseline difference in exploration participation. This model was an acceptable fit to data (Supplementary Fig. S2; Table 2). The intervention was positively associated with T1 exploration participation (*b* = 0.11, *SE* = 0.07, *p* = 0.02) and T1 exploration participation remained positively associated with T2 resolution (*b* = 0.31, *SE* = 0.05, *p* < 0.001). A multigroup model indicated no differences between minoritized and majoritized youth (Table 3).

Next, a reversed replication model was run to test the robustness of the hypothesized direction of effects in the conceptual model of change and was a good fit to data (Supplementary Fig. S3; Table 2). The intervention was positively associated with T1 resolution (*b* = 0.12, *SE* = 0.06, *p* = 0.02) and T1 resolution was in turn positively associated with T2 exploration participation (*b* = 0.30, *SE* = 0.07, *p* < 0.001). Further, a reversed replication model that also accounted for the intervention and control groups’ baseline difference in exploration participation was a good fit to data (Supplementary Fig. S4; Table 2), The associations between the intervention and T1 resolution (*b* = 0.18, *SE* = 0.06, *p* < 0.001) and T1 resolution and T2 exploration participation (*b* = 0.21, *SE* = 0.07, *p* < 0.001) remained positive. The respective multigroup analyses for the reversed replication models showed no differences between minoritized and majoritized adolescents (Table 3).

**Fig. 1:**
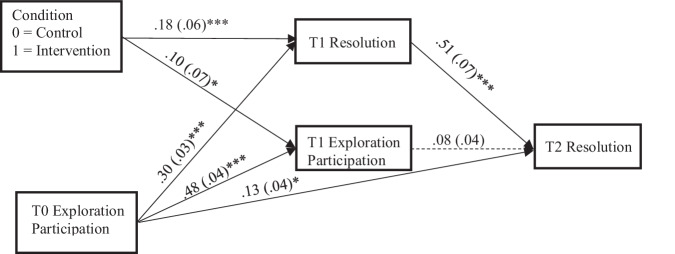
Stringent replication model. This model examined if the intervention was associated with T1 exploration participation and if T1 exploration participation was associated with T2 resolution, while controlling for T1 resolution. The model accounted for the observed baseline difference in exploration participation between the intervention and control group. T Time. Standardized estimates are presented. Solid lines indicate significant associations. *p < 0.05; ***p < 0.001

A stringent replication model (Fig.1/Supplementary Fig. S5) that controlled for T1 resolution and for the observed difference between the intervention and control group regarding baseline exploration participation was a good fit to data (Table 2). The intervention was positively associated with T1 exploration participation (*b* = 0.10, *SE* = 0.07, *p* = 0.02) and T1 resolution (*b* = 0.18, *SE* = 0.06, *p* < 0.001). However, T1 exploration participation was no longer associated with T2 resolution (*b* = 0.08, *SE* = 0.04, *p* = 0.11). On the other hand, T1 resolution (*b* = 0.51, *SE* = 0.07, *p* < 0.001) was positively associated with T2 resolution. Minoritized and majoritized youth did not differ in their paths (Table 3).

The next model expanded on the stringent replication model by including exploration search and displayed good fit indices (Supplementary Fig. S6; Table 2). The intervention was not associated with T1 exploration search (*b* = 0.06, *SE* = 0.06, *p* = 0.19) and T1 exploration search was not associated with T2 resolution (*b* = 0.07, *SE* = 0.06, *p* = 0.22). All other associations from the stringent replication model were unchanged. A multigroup model showed no difference between minoritized and majoritized youth (Table 3).

Next, private regard was included in the stringent replication model on an exploratory basis and was a good fit to data (Supplementary Fig. S7; Table 2). The intervention remained positively associated with T1 exploration participation, however, T1 exploration participation was not associated with T2 private regard (*b* = 0.07, *SE* = 0.06, *p* = 0.17). Furthermore, there was not a direct association between the intervention and T1 private regard (*b* = 0.09, *SE* = 0.10, *p* = 0.08). A multigroup model indicated that all paths were not equivalent for minoritized and majoritized youth (Table 3). To identify which of the paths were not equivalent, ad-hoc Wald χ^2^ test of the paths was conducted. Results from the Wald test indicated that baseline exploration participation was positively associated with T2 private regard for minoritized youth meanwhile there was no significant association for majoritized youth (*χ*^*2*^ = 9.04 (*df* = 1, *p* = < 0.01). Furthermore, T1 private regard was positively associated with T2 private regard for both groups, however, the association was stronger for majoritized youth (*χ*^*2*^ = 4.65 (*df* = 1, *p* =<0.05). All other paths were equivalent.

Finally, T1 and T2 centrality was also included in the conceptual model on an exploratory basis and was a good fit to data (Supplementary Fig. S8; Table 2). The intervention remained positively associated with T1 exploration participation, however, T1 exploration participation was not associated with T2 centrality (*b* = 0.05, *SE* = 0.05, *p* = 0.35). Furthermore, the intervention was positively associated with T1 centrality (*b* = 0.15, *SE* = 0.08, *p* = < 0.01). A multigroup model indicated that all paths could not be constrained to be equal for minoritized and majoritized youth (Table 3). Ad-hoc Wald χ^2^ test showed that the inequivalence of paths concerned the same two paths relating to private regard in the previous model (Supplementary Fig. S7).

### Trajectories of Change for Process Components

#### Exploration Participation

**Fig. 2:**
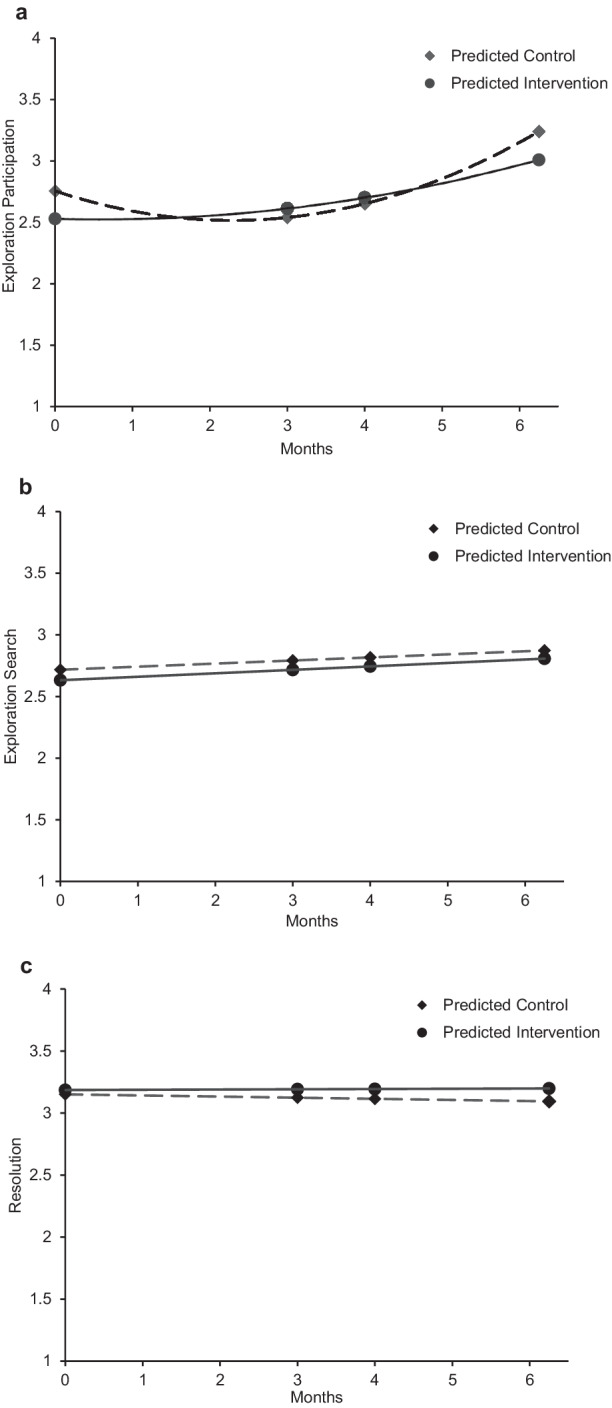
Trajectories of change for exploration participation, exploration search and resolution. Estimated trajectories of change for ethnic-racial identity processes by condition. **a** Exploration participation. **b** Exploration search. **c** Resolution. Solid black lines for intervention group. Dashed lines for control group. See Tables 5 and 6 for all slope estimates

The unconditional quadratic growth model was determined to be the best functional form for exploration participation (Table 4). The linear slope factor indicated that the adolescents’ average level of exploration participation initially increased, and the quadratic slope factor indicated a deceleration in this rate of change over time (Fig. 2; Table 5). There was variability in the initial levels of exploration participation in the sample, however, there was no variability in the linear or quadratic slope factors.

**Table 4:** Latent growth curve model comparison for exploration participation, exploration search, resolution, private regard and centrality

Model	*N*	χ^2^	df	χ^2^diff	Δ*df*	*p*	CFI	TLI	RMSEA (90% CI)	SRMR	AIC	BIC
Exploration participation												
Unconditional												
Linear	509	12.04	5				0.97	0.97	0.07 (0.02–0.13)	0.05	3479.33	3517.42
Quadratic	509	0.17	1	11.61	4	0.02*	1.00	1.02	0.00 (0.00–0.16)	0.00	3474.17	3529.19
Conditional												
Linear	509	25.92**	7				0.96	0.95	0.08 (0.03–0.12)	0.04	3479.37	3525.93
Quadratic	509	0.07	2	22.16	5	<0.001***	1.00	1.05	0.00 (0.00–0.36)	0.05	3465.44	3533.16
Exploration search												
Unconditional												
Linear	508	4.57	5				1.00	1.01	0.00 (0.00–0.08)	0.03	2817.58	2855.65
Quadratic	508	4.44*	1	1.06	4	0.90	0.99	0.95	0.09 (0.02–0.19)	0.02	2824.13	2879.12
Conditional												
Linear	509	8.42	7				1.00	1.02	0.00 (0.00–0.07)	0.03	2819.03	2865.59
Quadratic	509	0.28	2	6.97	5	0.22	0.30	−2.51	0.60 (0.31–0.94)	0.18	2820.08	2887.80
Resolution												
Unconditional												
Linear	509	2.22	8				1.00	1.02	0.00 (0.00–0.00)	0.03	2806.79	2832.19
Quadratic	509	0.76	4	1.45	4	0.84	1.00	1.03	0.00 (0.00–0.00)	0.02	2812.35	2854.68
Conditional												
Linear	509	6.54	10				1.00	1.18	0.00 (0.00–0.00)	0.03	2808.49	2842.35
Quadratic	509	1.56	5	4.69	5	0.45	1.00	1.61	0.00 (0.00–0.00)	0.05	2811.26	2866.28
Private regard												
Unconditional												
Linear	508	3.14	5				1.00	1.01	0.00 (0.00–0.07)	0.03	3774.67	3812.75
Quadratic	508	0.89	1	2.35	4	0.67	1.00	1.00	0.00 (0.00–0.13)	0.01	3779.00	3834.00
Conditional												
Linear	509	4.31	7				1.00	1.01	0.00 (0.00–0.07)	0.03	3777.91	3824.47
Quadratic	509	2.08	2	2.80	5	0.73	1.00	1.12	0.00 (0.00–0.32)	0.03	3783.81	3851.53
Centrality												
Unconditional												
Linear	509	6.78	5				1.00	1.00	0.03 (0.00–0.09)	0.04	3414.89	3452.99
Quadratic	509	0.07	1	6.64	4	0.16	1.00	1.01	0.00 (0.00–0.19)	0.00	3415.96	3470.98
Conditional												
Linear	509	13.75	7				0.98	0.97	0.07 (0.03–0.12)	0.05	3415.34	3461.90
Quadratic	509	1.88	2	11.03	5	0.05	0.94	0.63	0.22 (0.00–0.54)	0.08	3414.24	3481.96

Robust fit indices were used

*χ2diff* nested χ2 difference, *CFI* comparative fit index, *TLI* Tucker-Lewis index, *RMSEA* root mean square error of approximation, *SRMR* standardized root mean square residual, *CI* Confidence interval*, AIC* Akaike information criterion, *BIC* Bayesian information criterion

*p < 0.05; **p < 0.01; ***p < 0.001

**Table 5:** Unconditional latent growth curve models for ethnic-racial identity exploration and resolution

	Exploration participation		Exploration search		Resolution	
	*b*	*SE*	*b*	*SE*	*b*	*SE*
Factor means						
Intercept (*b*_*0*_)	2.40***	0.04	2.58***	0.03	3.20***	0.03
Linear slope (*b*_1_)	0.12***	0.02	0.03***	0.01	0.01	0.01
Quadratic slope (*b*_2_)	−0.01*	0.00				
Factor variances						
Intercept variance	0.58*	0.28	0.21***	0.05	0.26***	0.04
Linear slope variance	0.08	0.06	0.00	0.00	0.01*	0.00
Quadratic slope variance	0.00	0.00				
Factor covariances						
Intercept - linear slope	−0.12	0.13	0.00	0.01	−0.01*	0.01
Intercept - quadratic slope	0.01	0.01				
Linear slope - quadratic slope	−0.01*	0.00				

Unstandardized estimates presented

*SE* standard error

*p < 0.05; ***p < 0.001

In the conditional model for exploration participation, the intervention was negatively associated with exploration participation at baseline, i.e., with the model intercept, indicating that the intervention group started at a lower level of participation than the control group (Fig. 2; Table 6). The intervention was positively associated with the linear slope, indicating that the intervention group had a higher rate of initial change compared to the control group. The intervention was not associated with the quadratic slope. The conditional growth model was then run as a multigroup model, grouped by minoritization. A nested multigroup model comparison of an unconstrained model and a model in which the intervention effect on intercept and slopes was constrained to be equal for minoritized and majoritized adolescents showed no difference (Table 7).

**Table 6:** Conditional latent growth curve models for ethnic-racial identity exploration and resolution

	Exploration participation		Exploration search		Resolution	
	*b*	*SE*	*b*	*SE*	*b*	*SE*
Factor means						
Intercept	2.76***	0.13	2.72***	0.10	3.15***	0.10
Intervention	−0.23**	0.08	−0.09	0.06	0.04	0.06
Linear slope	−0.21	0.13	0.03	0.02	−0.01	0.02
Intervention	0.19**	0.07	0.00	0.01	0.01	0.01
Quadratic slope	0.05	0.03				
Intervention	−0.03	0.02				
Factor variances						
Intercept variance	0.60*	0.27	0.21***	0.05	0.26***	0.04
Linear slope variance	0.08	0.06	0.00	0.00	0.01*	0.00
Quadratic slope variance	0.00	0.00				
Factor covariances						
Intercept - linear slope	−0.13	0.13	0.00	0.01	−0.01*	0.01
Intercept - quadratic slope	0.01	0.00				
Linear slope - quadratic slope	−0.01	0.01				

Unstandardized estimates presented. Condition coded as 0 = control group and 1 = intervention

*SE* standard error

*p < 0.05; **p < 0.01; ***p < 0.001

**Table 7:** Latent multigroup growth curve model comparison for exploration participation, exploration search, resolution, private regard and centrality

Model	*N*	χ^2^	df	χ^2^diff	Δ*df*	*p*	CFI	TLI	RMSEA (90% CI)	SRMR	AIC	BIC
Exploration participation												
Unconstrained	471	7.50	8				1.00	1.11	0.00 (0.00–0.29)	0.08	3239.61	3355.95
Constrained	471	9.98	11	2.48	3	0.48	1.00	1.08	0.00 (0.00–0.16)	0.07	3235.94	3339.81
Exploration search												
Unconstrained	471	19.13	14				1.00	1.06	0.00 (0.00–0.08)	0.05	2591.65	2683.05
Constrained	471	20.93	16	1.73	2	0.42	1.00	1.06	0.00 (0.00–0.06)	0.05	2589.20	2672.30
Resolution												
Unconstrained	471	22.77	21				1.00	1.10	0.00 (0.00–0.04)	0.06	2598.92	2661.24
Constrained	471	25.69	23	3.19	2	0.20	1.00	1.10	0.00 (0.00–0.00)	0.05	2598.02	2652.03
Private regard												
Unconstrained	471	6.39	14				1.00	1.24	0.00 (0.00–0.00)	0.03	3568.30	3659.71
Constrained	471	7.86	16	1.56	2	0.46	1.00	1.21	0.00 (0.00–0.00)	0.03	3565.77	3648.86
Centrality												
Unconstrained	471	19.32	14				1.00	1.05	0.00 (0.00–0.07)	0.05	3223.06	3314.46
Constrained	471	19.61	16	0.48	2	0.79	1.00	1.04	0.00 (0.00–0.06)	0.05	3219.51	3302.61

Group coded as 0 = minoritized and 1 = majoritized.

*Constrained*, equality constraints on intervention effect on intercepts and slopes by minoritization. Robust fit indices were used*. χ2diff*, nested χ2 difference; *CFI*, comparative fit index; *TLI*, Tucker-Lewis index; *RMSEA*, root mean square error of approximation; *SRMR*, standardized root mean square residual; *CI*, Confidence interval*, AIC*, Akaike information criterion; *BIC*, Bayesian information criterion

Regarding group means post-intervention, the intervention and control group did not differ in the mean level of exploration participation at T1 (*t*(429) = − 0.94, *p* = 0.17, Cohen’s *d* = −0.09, Intervention: *M* = 2.70, *SD* = 0.78, Control: *M* = 2.63, *SD* = 0.78) or at T2 (*t*(427) = − 0.42, *p* = 0.68, Cohen’s *d* = −0.04; Intervention: *M* = 2.76, *SD* = 0.84, Control: *M* = 2.72, *SD* = 0.77; see preliminary analyses for baseline comparisons).

#### Exploration Search

The unconditional linear growth model was determined to be the best functional form for exploration search (Table 4). The linear slope factor indicated that the adolescents’ average level of exploration search increased over time (Fig. 2; Table 5). There was variability in the initial level of exploration search among adolescents but no variability in their trajectories of change. In the conditional model, the intervention was not associated with exploration search at baseline nor was it associated with the linear slope, indicating that the intervention and control groups did not vary in their initial level of exploration search or in their trajectories of change (Fig. 2; Table 6). A multigroup model showed no difference in the intervention effect for minoritized and majoritized adolescents (Table 7).

Further, the intervention and control groups did not differ in their post-intervention mean level of exploration search at T1 (*t*(430) = − 0.04, *p* = 0.48, Cohen’s *d* = −0.00, Intervention: *M* = 2.64, *SD* = 0.66, Control: *M* = 2.64, *SD* = 0.69). The control group had a higher level of exploration search than the intervention group at T2 (*t*(425) = 1.99, *p* = 0.048, Cohen’s *d* = 0.19; Intervention: *M* = 2.69, *SD* = 0.73, Control: *M* = 2.82, *SD* = 0.61).

#### Resolution

The unconditional linear growth model was determined to be the best functional form for resolution (Table 4). The linear slope factor indicated that the adolescents’ average level of resolution did not change over time (Fig. 2; Table 5). The visualized average trajectory for resolution suggested a possible ceiling effect, with a relatively high and stable average level of resolution over time. However, there was variability in the initial level of resolution and in the adolescents’ trajectories of change. In the conditional model, the intervention was not associated with resolution at baseline or with the linear slope, indicating that the intervention and control groups did not vary in their initial level of resolution or in their trajectories of change (Table 6). A multigroup model showed no difference in the intervention effect for minoritized and majoritized adolescents (Table 7).

The intervention group had a higher mean on resolution than the control group at T1 (*t*(367) = −2.47, *p* = 0.01, Cohen’s *d* = −0.24, Intervention: *M* = 3.31, *SD* = 0.58, Control: *M* = 3.15, *SD* = 0.70), but not at T2 (*t*(430) = −1.18, *p* = 0.12, Cohen’s *d* = −0.11, Intervention: *M* = 3.31, *SD* = 0.66, Control: *M* = 3.23, *SD* = 0.66).

### Trajectories of Change for Content Components

#### Private Regard

**Fig. 3:**
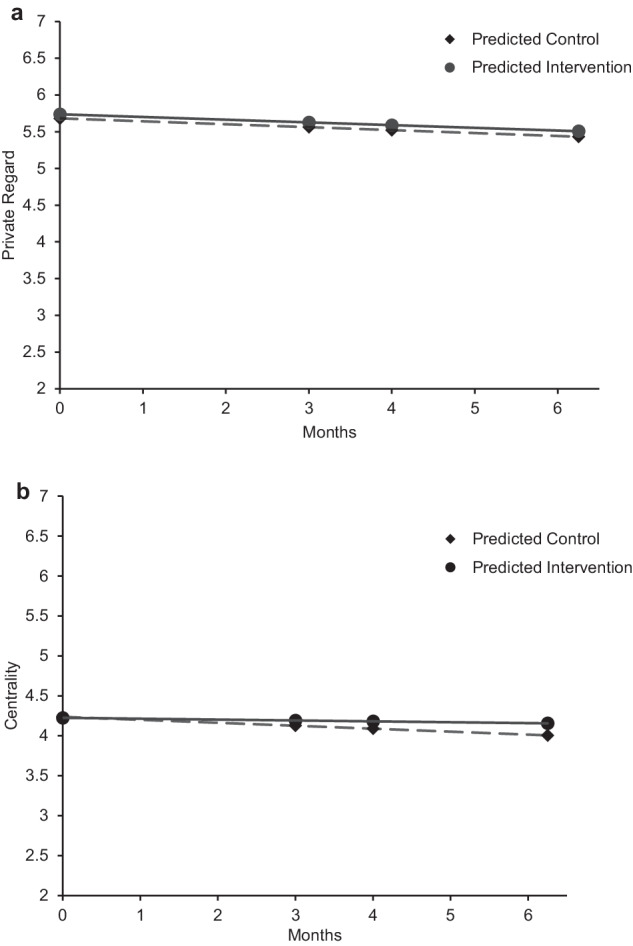
Trajectories of change for private regard and centrality. Estimated trajectories of change for ethnic-racial identity content by condition. **a** Private regard. **b** Centrality. Solid black lines for intervention group. Dashed lines for control group. See Tables 8 and 9 for all slope estimates

The unconditional linear growth model was determined to be the best functional form for private regard (Table 4). The linear slope factor indicated that the adolescents’ average level of private regard decreased over time (Table 8). There was variability in both the initial levels of private regard and in adolescents’ trajectories of change. In the conditional model, the intervention was not associated with private regard at baseline or with the linear slope (Fig. 3; Table 9), indicating that the intervention and control groups did not vary in their initial level of private regard or in their average trajectories of change. A multigroup model showed no difference in the intervention effect for minoritized and majoritized adolescents (Table 7).

**Table 8:** Unconditional latent growth curve models for ethnic-racial identity private regard and centrality

	Private regard		Centrality	
	*b*	*SE*	*b*	*SE*
Factor means				
Intercept (*b*_*0*_)	5.77***	0.04	4.21***	0.04
Linear slope (*b*_1_)	−0.03***	0.01	0.01	0.01
Factor variances				
Intercept variance	0.49***	0.08	0.59***	0.07
Linear slope variance	0.01**	0.00	0.01	0.00
Factor covariances				
Intercept and linear slope	−0.00	0.01	−0.03***	0.01

Unstandardized estimates presented

*SE* standard error

**p < 0.01; ***p < 0.001

**Table 9:** Conditional latent growth curve models for ethnic-racial identity private regard and centrality

	Private Regard		Centrality	
	*b*	*SE*	*b*	*SE*
Factor means				
Intercept	5.68***	0.14	4.24***	0.14
Intervention	0.06	0.08	−0.01	0.08
Linear slope	−0.04	0.03	−0.04	0.03
Intervention	0.00	0.02	0.03	0.02
Factor variances				
Intercept variance	0.48***	0.08	0.59***	0.07
Linear slope variance	0.01**	0.00	0.01	0.00
Factor covariances				
Intercept - linear slope	−0.00	0.01	−0.04**	0.01

Unstandardized estimates presented. SE standard error. Condition coded as 0 = control group and 1 = intervention

**p < 0.01; ***p < 0.001

Further, the intervention and control groups did not differ in their mean level of private regard at T1 (*t*(428) = −0.96, *p* = 0.34, Cohen’s *d* = −0.09, Intervention: *M* = 5.75, *SD* = 0.92, Control: M = 5.66, *SD* = 0.99) or at T2 (*t*(424) = −0.71, *p* = 0.48, Cohen’s *d* = −0.07, Intervention: *M* = 5.67, *SD* = 1.00, Control: *M* = 5.60, *SD* = 1.06).

#### Centrality

The unconditional linear model was determined to be the best functional form for centrality (Table 4). The linear slope factor indicated that the adolescents’ average level of centrality did not change over time (Table 8). In the conditional model, the intervention was not associated with centrality at baseline or with the linear slope, indicating that the intervention and control groups did not vary in their initial level of centrality or in their average trajectories of change (Fig. 3; Table 9). A multigroup model showed no difference in the intervention effect for minoritized and majoritized adolescents (Table 7).

Finally, the intervention group had a higher mean of centrality than the control group at T1 (*t*(429) = −2.25, *p* = 0.03, Cohen’s *d* = −0.22, Intervention: *M* = 4.31, *SD* = 0.90, Control: *M* = 4.11, *SD* = 0.91) but not at T2 (*t*(426) = −0.46, *p* = 0.65, Cohen’s *d* = −0.05, Intervention: *M* = 4.28, *SD* = 0.85, Control: *M* = 4.25, *SD* = 0.72).

## Discussion

Ethnic-racial identity is an identity domain that is particularly relevant for youth growing up in ethno-racially diverse societies. However, most previous research on ethnic-racial identity development has been conducted in the U.S. and there is thus a need for better understanding of the topic in understudied yet increasingly ethno-racially diverse contexts, such as Sweden. This study examined whether a school-based intervention, the Identity Project, positively impacted ethnic-racial identity processes exploration and resolution, and explored potential effects on ethnic-racial identity content components private regard and centrality among 10^th^ grade students. The findings showed patterns of both stability and change, and primarily indicated an effect on ethnic-racial identity processes.

### The Conceptual Model of Ethnic-racial Identity Change

Regarding the first research question, results from the path analyses partially support the hypothesized intervention effects. The replication model did not detect the hypothesized positive effect of the intervention on post intervention (T1) exploration participation. However, all models that accounted for the baseline (T0) difference between the intervention and control group in exploration participation indicated a positive intervention effect on youth exploration participation. This finding aligns with those from the U.S. (Umaña-Taylor et al., 2018), Germany (Juang et al., 2020), and Italy (Ceccon et al., 2023).

Nevertheless, the current findings do not support the hypothesized sequential increase in exploration participation and resolution, as there was no association between post-intervention (T1) exploration participation and later (T2) resolution. Instead, the findings indicate that the intervention had a simultaneous positive effect on T1 exploration participation and T1 resolution, and that T2 resolution was in large part driven by the prior level of resolution. The observed simultaneous effect on exploration participation and resolution could be beneficial for youth compared to prolonged ethnic-racial identity exploration without resolution (Wantchekon et al., 2021). Increases in ethnic-racial identity exploration is associated with a heightened risk for negative impacts of discrimination when youth are not also attaining a sense of resolution (Yip et al., 2019). This is especially the case for minoritized youth. The fact that the intervention impacted exploration participation and resolution simultaneously is thus encouraging.

Further, while T1 exploration participation was not related to T2 resolution, T1 resolution was indeed positively related to T2 exploration participation (reversed replication models). This finding supports previous research suggesting that youth may continue to explore their ethnic-racial identities after periods of feeling resolved (Sladek et al., 2023). Taken together, the path analyses suggest a dynamic development of, and relationship between, exploration participation and resolution. Thus, a turn to dual cycle identity formation models could be of interest to understand how youth move within and between facets of ethnic-racial identity (Crocetti, 2017). In such models, identity develops in two related yet distinct cycles.

Contrary to the hypothesis that the intervention affects both types of exploration, the intervention acted on youth exploration participation but not search. There could be several reasons for this. First, the intervention is specifically built on participatory activities and consequently it may only act on the form of exploration it was designed to target (Umaña-Taylor et al., 2018), as the intervention activities and aims are more clearly linked to items in the Ethnic Identity Scale-Brief (Douglass & Umaña-Taylor, 2015) compared to items in the Multigroup Ethnic Identity Measure (Phinney, 1992). Furthermore, the scales focus on the extent to which youth engaged in exploration items during the last 30 days, and an intervention effect on participation was most noticeable partly in relation to a period when adolescents were engaging in specific participatory activities as part of the intervention. There is thus a risk that the increased participation at T1 is a sign of a successful intervention implementation rather than identity change. However, the mean level of youth participation post-intervention seemingly stayed stable, suggesting that the adolescents, on average, maintained a higher level of ethnic-racial identity exploration even after the intervention.

The exploratory assessment of private regard and centrality within the conceptual model of change indicated that the intervention did not have any direct or indirect effect on private regard, however, the intervention was positively associated with T1 centrality. Despite these results, the findings concerning content components, especially centrality, need to be interpreted with great caution as confirmatory factor analyses indicate that the scales did not work reliably in the current study. Similar reliability levels were found for the centrality scale in U.S.- based studies with youth with varying ethnic-racial self-identifications (e.g., Sladek et al., 2020). Taken together, these findings suggest that the widely used adaptation (changing “Black” to “ethnicity”) of the private regard and centrality subscales needs further scrutiny (Moffit & Rogers, 2022).

### Trajectories of Change for Ethnic-racial Identity Process and Content

Regarding the second research question, the hypothesis concerning increased process components over time as an effect of the intervention was only supported for exploration participation but not for search or resolution, indicating that the intervention had a narrow impact over time on its primary target construct. Nevertheless, the average level of search did increase over time for the whole sample irrespective of study condition. Search could thus be understood as a normative developmental process of ethnic-racial identity that occurs during adolescence (Phinney, 1990). Search may have been triggered by filling out the study questionnaire (Syed et al., 2011) or by a heightened salience of ethnic-racial identity due to the students’ recent transitions from secondary school to high school (French et al., 2006). Finally, the average level of resolution did not change over time, and both the intervention and control groups had a high level of resolution from baseline and throughout. Considering that the current study population was older than adolescents in previous evaluation studies (e.g. Ceccon et al., 2023), they may have already followed an age-based developmental process in which youth are more likely to have higher levels of resolution later in adolescence (Umaña-Taylor et al., 2014). However, the intervention group did have a higher level of resolution than the control group at T1, when they had just received the intervention. In sum, the findings speak to both elements of change and relative stability over time, in youth ethnic-racial identity development (Syed et al., 2007).

The exploratory assessment of content components over time indicated that private regard decreased over time and that this decrease was unrelated to the intervention. It is thus unclear why such a decrease over time occurred. Prior studies have reported that private regard may change noticeably from day to day and that it is sensitive to one’s social environment (Seaton & Iida, 2019). Nonetheless, it is noted that both the intervention and control group started and remained at high average levels of private regard throughout the study. Moreover, the average trajectory for centrality showed no change over time and the intervention was not associated with this trajectory. Once again, the poor reliability of scales, especially concerning centrality, limit the possible inferences that can be drawn from these findings.

### Minoritization and Response to the Intervention

Regarding the third research question, minoritization did not moderate the path models that only included process components exploration and resolution. For models that included private regard and centrality, all paths except for two were equal for minoritized and majoritized youth. These were however not related to the intervention and thus, all paths associated with the intervention were equivalent throughout. Furthermore, the intervention effect on intercepts and slopes of all process and content components were equivalent for minoritized and majoritized youth. This is in line with the goal of the intervention to be equally effective for youth regardless of their backgrounds (Umaña-Taylor et al., 2018).

Although the intervention did not have differential effects by minoritization, minoritized youth did start at higher levels on both process and content components compared to majoritized youth, which is consistent with previous research suggesting higher salience of ethnic-racial identity for minoritized populations (Rivas-Drake et al., 2014). Although ethnicity and race related themes tend to be conceptualized as uncomfortable, taboo, or even irrelevant in some Swedish contexts (Hübinette et al., 2023), it is noteworthy that adolescents in the current study reported relatively high averages on both exploration participation and resolution. The mean level of these process components at baseline, i.e., even before intervention, were at similar or higher levels than the U.S. intervention study average (Umaña-Taylor et al., 2018). This speaks to a developmental salience of ethnic-racial identity in Sweden.

Importantly, the construction of minoritization and majoritization based on familial migration histories in Swedish contexts entails limitations. Such categorization applies a dichotomy between the constructed groups and does not capture variability in possible self-identifications within each group. Moreover, it does not capture minoritization based on prevalent racialization processes in Sweden, such as assumptions of “Swedishness” often being related to whiteness (Adolfsson, 2024). Thus, individuals can be born in Sweden to parents born in Sweden and still be ethno-racially minoritized due to whiteness or other markers such as names. Similarly, there are those who themselves or their parents are born outside of Sweden or Europe who are ethno-racially majoritized due to whiteness. The problem at hand is that birthplace or parental birthplace does not inform how youth de-facto are racialized in their daily lives. Nevertheless, familial migration history is one aspect of minoritization that is used as a proxy in Sweden.

### Implications

The current study highlights that a school-based intervention focused on creating a space for youth to have structured and active engagement in discussions and critical reflection on the constructs of race and ethnicity, with peers, and with support, yielded initial simultaneous increases in youth ethnic-racial identity exploration participation and resolution. Although the collective findings in this study highlight both elements of stability and change, the co-occurrence of exploration participation and resolution provide a favorable outset to interrogate whether these process components of ethnic-racial identity can be mechanisms by which positive psychosocial adjustment can be attained for youth in Sweden. Furthermore, the findings highlight that ethnic-racial identity is not unique to the social fabrics of the U.S., but rather, youth across the world are engaging in similar processes, adding to the cumulative knowledge about youth ethnic-racial identity development.

Further, the differential effect of the intervention on exploration participation and exploration search underscores the importance of studying facets of ethnic-racial identity holistically, and separately, to facilitate a more nuanced understanding of the components. As this was the first evaluation study examining the conceptual model of change in a Swedish context, replication and refinement of assessed models, and measures, can strengthen possible inferences.

### Strengths and Limitations

The current study has both strengths and limitations to consider when drawing inferences based on results. Strengths include design aspects of the study such as utilizing a wait-list control group design, class-wise randomization, multiple follow-up measurements, and a large sample size. The stringent assessment of preregistered conceptual models and adherent hypotheses further strengthen the study. Moreover, change trajectories of all key study variables are included, representing both process and content components of the multidimensional meta-construct ethnic-racial identity (Umaña-Taylor et al., 2014).

Nevertheless, limitations to consider pertain to the intervention and wait-list control group attending the same schools, which entails a risk that the wait-list control group may have been privy to some parts of the intervention beforehand (Aycock et al., 2018). However, the current design ensured that all students could receive intervention and allowed for a comparable control group within the same academic year. Further, a limitation due to the wait-list control group design was that the intervention group only had a comparable control at T0, T1 and T2 since the control group had also received the intervention by T3. This was not an issue for the path models which utilized time point T0, T1 and T2. It was however an issue when examining change trajectories from T0 to T3, where the control group’s observed data was treated as missing at T3. Nevertheless, because this was a known pattern of missingness, it was accounted for in the conditional growth curve models with the inclusion of intervention as a predictor of intercepts and slopes. Moreover, robustness checks using the first three waves of data supported the reported findings.

Another limitation of the study is that the randomization of classrooms into an intervention and control group did not work as expected on the key study variable exploration participation. Unfortunately, the intervention group had a lower level of participation than the control group. This problem was handled through the preregistered plan to account for such potential differences between intervention and control group in data analysis, by including T0 exploration participation in the assessment of the conceptual model change.

Furthermore, the measures used for key study variables were not validated for Swedish school contexts, with most issues adherent to the centrality scale. Additionally, effect sizes in the current study were small, and in line with previous research indicating small effect sizes to be the norm regarding evaluation of universal interventions (Kraft, 2020). Furthermore, the intervention was implemented during the Covid-19 pandemic, which for example could entail abruptly canceled or rescheduled classes. These types of structural changes, as well as potential facilitator effects, questions of fidelity (concerning researchers as well as for example students partaking in some activities and not others) could all be variations that may impact observed effects of the intervention. Finally, the intervention was evaluated as an eight-lesson package making it difficult to decipher which parts were more or less effective. Since the intervention needs time and personnel, which schools may struggle to allocate, future directions include a focus on identifying potential moderators of the intervention.

## Conclusion

Ethnic-racial identity is a central developmental task that is particularly relevant for youth growing up in ethno-racially diverse societies. Given that much of the knowledge about ethnic-racial identity and how it develops has been conducted in the U.S., there is a need for a better understanding of the topic in understudied yet increasingly ethno-racially diverse contexts, such as Sweden. This study examined whether a school-based intervention could prompt ethnic-racial identity processes, exploration and resolution, and explored potential effects on ethnic-racial identity content, private regard and centrality, among adolescents. The findings showed patterns of both stability and change, and indicated that an intervention focused on discussions and critical reflection on the constructs of race and ethnicity, with peers, and with support, yielded a positive and simultaneous effect on exploration participation and resolution, and indicated a positive effect on exploration participation over time. At the same time, the collective findings suggest that the intervention is narrowly tailored towards exploration participation and, to a lesser extent, resolution. The findings have important implications for identity theory and research as they add to prior research proposing that exploration and resolution may have a simultaneous and/or reciprocal relationship rather than a linear relationship. Thus, the results highlight a need for dynamic approaches to understanding how youth might move within and between domains of ethnic racial identity. The findings also highlight that ethnic-racial identity is not unique to the social fabrics of the U.S., but rather, young people in European contexts are engaging in similar processes.
